# Occurrence and Risk Assessment of PAHs in Surface Sediments from Western Arctic and Subarctic Oceans

**DOI:** 10.3390/ijerph15040734

**Published:** 2018-04-12

**Authors:** Fajin Chen, Yan Lin, Minggang Cai, Jingjing Zhang, Yuanbiao Zhang, Weiming Kuang, Lin Liu, Peng Huang, Hongwei Ke

**Affiliations:** 1 Guangdong Province Key Laboratory for Coastal Ocean Variation and Disaster Prediction, Guangdong Ocean University, Zhanjiang 524088, China; fjchen04@163.com (F.C.); penghuang@xmu.edu.cn (P.H.); 2 Fujian Provincial Key Laboratory for Coastal Ecology and Environmental Studies, Xiamen University, Xiamen 361102, China; yanlin@stu.xmu.edu.cn (Y.L.); mgcai@xmu.edu.cn (M.C.); 3 College of Ocean and Earth Sciences, Xiamen University, Xiamen 361002, China; zjj@sio.org.cn (J.Z.); 22320152201027@stu.xmu.edu.cn (L.L.); 4 State Key Laboratory of Marine Environmental Science, Xiamen University, Xiamen 361002, China; 5 Third Institute of Oceanography, State Oceanic Administration, Xiamen 361005, China; zhangyuanbiao@tio.org.cn (Y.Z.); kuangweiming@tio.org.cn (W.K.)

**Keywords:** polycyclic aromatic hydrocarbons (PAHs), sediments, Bering Sea, Chukchi Sea, Canadian Basin, source apportionment, ecological and health risk assessment

## Abstract

In the fourth Chinese National Arctic Research Expedition (from July to September, 2010), 14 surface sediment samples were collected from the Bering Sea, Chukchi Sea, and Canadian Basin to examine the spatial distributions, potential sources, as well as ecological and health risk assessment of polycyclic aromatic hydrocarbons (PAHs). The ∑PAH (refers to the sum of 16 priority PAHs) concentration range from 27.66 ng/g to 167.48 ng/g (dry weight, d.w.). Additionally, the concentrations of ∑PAH were highest in the margin edges of the Canadian Basin, which may originate from coal combustion with an accumulation of Canadian point sources and river runoff due to the surface ocean currents. The lowest levels occurred in the northern of Canadian Basin, and the levels of ∑PAH in the Chukchi Sea were slightly higher than those in the Being Sea. Three isomer ratios of PAHs (Phenanthrene/Anthracene, BaA/(BaA+Chy), and LMW/HMW) were used to investigate the potential sources of PAHs, which showed the main source of combustion combined with weaker petroleum contribution. Compared with four sediment quality guidelines, the concentrations of PAH are much lower, indicating a low potential ecological risk. All TEQ_PAH_ also showed a low risk to human health. Our study revealed the important role of the ocean current on the redistribution of PAHs in the Arctic.

## 1. Introduction

Polycyclic aromatic hydrocarbons (PAHs) are a class of widely distributed organic compounds originating from incomplete combustion and pyrolysis of carbonaceous materials [1]. The concentrations of PAHs should be seriously controlled due to the fact that PAHs can propel the formation of carcinogenic and malformed molecules in the living organisms, and some high molecular weight PAHs have bio-accumulation characteristics in the food chain [2,3,4]. Simultaneously, scientists justified that during the formation, transportation, transformation, and degradation processes [5], PAHs can enter into human bodies through skin, breath, and alimentary canal, which contribute to an extreme threat to our health [6]. In addition, PAHs tend to remain in the environment for a long time due to their chemical stability, and they can migrate globally from areas of human activity through distillation and condensation [7]. That is why PAHs are the principal monitored contaminates and have been an increasing environmental concern in the last three decades.

PAHs could be derived from natural and mainly anthropogenic sources [8,9]. Natural sources include volcanic activity and spontaneous formation by plants and microbes, while anthropogenic PAHs may be generated from fossil fuel combustion, municipal and industrial effluents, creosote, oil spills, urban and agricultural runoff, asphalt production, waste incineration, deposition, and transportation of aerosols [5,10]. Unlike other POPs that has been legally banned from use in many countries, PAHs are inevitably emitted into the environment through these ways. PAHs have the characteristic of strong hydrophobicity and lipophilicity, which provide them with a strong affinity for particulate matters and sediments, thus their concentrations in sediment are higher than those in the overlying water column [11,12]. For remote pelagic environments like the North Pacific and Arctic Oceans, particle settling could be a significant removal process for hydrophobic chemicals [13]. Marine sediments play an important role in acting as long-term sinks for many anthropogenic contaminants involving PAHs and organic compounds, which have caused worldwide concern as toxic conservative contaminants [14,15,16,17,18,19,20]. Homogenously, contaminants in sediments can release to the seawater through suspension of sediments. That means that sediments are not merely significant receivers, but could also act as secondary sources of these persistent anthropogenic pollutants and greatly affect their fate in the environment [21,22]. Moreover, the levels and occurrence of pollutants in surface sediments can provide information on recent depositions .

The Arctic Ocean is no longer a pristine environment free of anthropogenic contaminants. PAHs in the Arctic region may originate from low or mid-latitude areas through atmospheric transportation, which is called the “grasshopper effect” [23]. Continental sources of PAHs via long-range transport from mid-latitudes have been demonstrated by several modeling studies [24,25]. Surface ocean currents are also considered to provide significant transfer routes to the Arctic due to ongoing deposition and gaseous exchange between the atmosphere and seawater [26]. On the other hand, climate change perturbations are also driving increased coastal erosion and permafrost thawing in the Arctic. Therefore, petrogenic PAHs are increasing due to transport over long distances via turbidity currents, as well as the offshore transport of sediment-laden ice from the coastal shelf areas and subsequent export between the shelves and the central Arctic Ocean [27]. Therefore, the Arctic acts as a final sink for semi-volatile pollutants. However, it has been suggested that, as primary sources of POPs are reduced, then remote areas like the Arctic Ocean may play a role as a secondary source, resulting in the re-emission of POPs [28].

The continental margins of the Chukchi and Bering Seas are some of the largest margins in the world and they have several unique characteristics that distinguished them from the other margins in the Arctic. Firstly, the region is bounded in the east and west by continents, as opposed to the rest of the arctic margins, which have land to the south and open ocean to the north. In addition, there are several sharp parameters such as nutrients across and along the shelf and they also have lower sea ice cover. Most importantly, their primary productivity is among the highest regions of the world margins, with Bering Sea supporting large bodies of commercial production. From this perspective, the Arctic shelf sediments are potentially important when considering human and ecological exposure via marine (pelagic) food webs (e.g., phytoplankton → zooplankton → Arctic cod → ringed seal).

Over the past three decades, PAHs have been monitored mainly in heavily contaminated coastal regions and polluted industrialized zones [2,17,29,30,31,32,33], while investigations in the remote pelagic environments like the North Pacific and Arctic Oceans are sparse as the harsh climate and environment create difficulties for sampling. Despite this, scientists have done a relatively great job concerning POP research in the air and surface water of the Arctic and Subarctic Oceans [28,31,34,35,36,37,38,39,40]. However, there are relatively few studies that have measured concentrations in deep ocean waters or bottom sediments, with some marine sediment studies being limited to coastal area such as the mid- and western Russian Arctic [41,42]; Norwegian Arctic [43,44,45]; Mackenzie shelf area [46]; Saglek Bay, Labrador, Canada [47]; and the Gulf of Alaska [48]. Therefore, for PAHs, the role of sediments in the western Arctic, subarctic oceans, and surrounding deep sea as an exchanging compartment with water and/or permanent sink is not fully understood and requires investigation. The aim of this study was to evaluate the levels, spatial distribution patterns, potential sources, and potential ecological and health risk of PAHs in surface sediments from the Bering Sea, Chukchi Sea, and Canadian Basin.

## 2. Sampling Strategy and Methods

### 2.1. Study Area and Sample Collection

**Figure 1:**
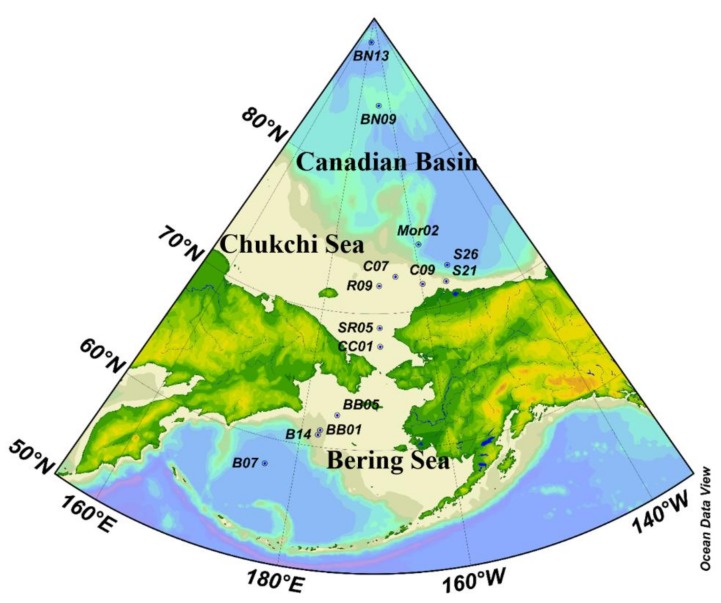
The sample stations in the study area.

Samples were collected from Bering Sea, Chukchi Sea, and Canadian Basin based on the fourth Chinese National Arctic Research Expedition from July to September 2010, onboard the R/V Xuelong (Snow Dragon). A total of 14 surface sediment samples (0–2 cm) were collected by a grab sampler (Figure 1). Detailed information about the location and depth were given in Table S1 in the supporting information. The surface sediment samples were transferred into 450 °C pre-combustion aluminum foil containers, and packaged them with aluminum foil again and marked with the station information. Then all samples were stored at −20 °C until further analysis.

### 2.2. Analysis of PAHs

#### 2.2.1. Pre-Treatment of Sediments, PAHs Extraction, and Cleanup

After transported to the laboratory, the sediment samples were freeze-dried and homogenized with a mortar and pestle, then sieved through an 80 mesh stainless steel sieve prior to analysis. Determination of surface sediment granulometry was measured using a laser particle size analyzer (Mastersizer 2000, Malverm, UK).

The sediment extraction was performed with accelerated solvent extractor (ASE, Thermo Co., Sunnyvale, CA, USA). 10 g of each sample were weighed, and then transferred into cells, spiked with 100 μL internal surrogate standard mixtures (Naphthalene-*d*_8_, Acenaphthene-*d*_10_, Chrysene-*d*_12_, Perylene-*d*_12_, Phenanthrene-*d*_10_). All samples were extracted with 100 mL dichloromethane (DCM) and 40 mL anhydrous sodium sulfate (Na_2_SO_4_), and ASE conditions were set up as follows: oven temperature: 100°C; pressure: 1500–2000 psi; static time: 5 min after 5 min pre-heat equilibration; flush volume: 60% of the cell volume; nitrogen purge: 60 s; static cycles: 2. The ASE extracts were collected in round bottomed flasks with funnels, which had been rinsed by DCM and filled with Na_2_SO_4_. The collected fluent was evaporated to approximately 1 mL after solvent exchange with hexane.

The purifying system was prepared by 500 mg/6 mL neutral Al_2_O_3_ SPE column (Sinopharm Chemical Reagent Co., Ltd., Shanghai, China) and 1000 mg/6 mL silica gel column (Ji Deyuan Reagent Factory, Qingdao, China). After cleaning the system with 5 mL mixture of pentane and DCM (1:1, *v*/*v*) and 5 mL hexane, targets were eluted with 15 mL mixture of pentane and DCM (1:1, *v*/*v*). A new concentration step was performed to a final volume of 1 mL with a gentle nitrogen stream. Finally, internal standard solution, 50 μL of pyrene-*d*_10_, was added to the extract before instrumental analysis.

#### 2.2.2. Instrumental Analysis of PAHs

All samples were analyzed for the following 16 PAHs in this study: naphthalene (Nap), acenaphthylene (Acep), acenaphthene (Acp), fluorine (Flu), phenanthrene (Phe), anthracene (Ant), fluoranthene (FLR), pyrlene (PYR), benzo[a]anthracene (B(a)A), chrysene (Chr), benzo[b]fluoranthene (B(b)F), benzo[k]fluoranthene (B(k)F), benzo(a)pyrene (B(a)P), dibenzo[a,h]anthracene (DaA), benzo[ghi]perylene (B(g)P), and indeno[1,2,3-cd]pyrene (InP).

PAH quantification was achieved by gas chromatography-mass spectrometry (GC-MS) using an HP5-MS capillary column (30 m length, 0.25 mm inner diameter, 0.25 μm film thickness; J & W Scientific Inc., Folsom, USA), with helium as carrier gas. The column oven was programmed from 60 °C to 150 °C at a rate of 15 °C/min, then from 150 °C to 220 °C at a rate of 5 °C/min, and finally from 220 °C to 300 °C at a rate of 10 °C/min with a holding time of 10 min.

#### 2.2.3. Quality Assurance and Quality Control

Blanks procedure was performed as mentioned above. The blank concentration was very low for PAHs. The concentration levels were obtained by subtracting the blanks from the values measured by chromatograms. Detection limits were quantified as the mean concentration in the field blank plus three times the standard deviation (0.012 ng g^−1^ for PAHs). Recoveries were determined for all samples by spiking with the surrogate standards prior to extraction. The mean recovery (%) of five surrogates range was 73.9%. The mean recoveries obtained for Naphthalene-*d*_8_ was 77.05%, Acenaphthene-*d*_10_ was 79.42%, Phenanthrene-*d*_10_ was 76.95%, Chrysene-*d*_12_ was 85.10%, whereas for Perylene-*d*_12_ the mean recovery (%) was 82.44%. Furthermore, the spike recoveries (%) were from 71.7% to 104.5%.

## 3. Results and Discussion

### 3.1. Sediment Properties (Grain Size)

All the grain sizes of the sediment were displayed in Table S2. According to the level of φ, the grain sizes of samples were classified into four parts, clay (φ > 8), silt (4 < φ < 8), gravel (2 < φ < 4), and sand (0 < φ < 2). Sediments in all study areas were mainly contributed by silt which ranged from 50.93% to 77.14%. Furthermore, the sediments contained relatively low sand which just were consisted of 1.465 ± 1.465%. Apparently, the highest percent of silt was discovered in station R09, while station BN09 had the lowest percent, indicating that the larger the sediment grain size, the lower the TOC content and the smaller the amount of adsorbed PAHs. In Bering Sea sediments, gravel contributed more than clay, while the exact opposite condition was found in the Canadian Basin, and that in Chukchi Sea sediments gravel was slightly more than clay. In general, the grain size properties of the study sediments showed no obvious geographic differences. Moreover, there were no strong correlations between grain size values and PAHs concentrations (Table S3), indicating the distribution pattern of PAHs concentration was mainly depended on their original sources.

### 3.2. Concentration and Composition

#### 3.2.1. PAHs Levels and Compositions

**Figure 2:**
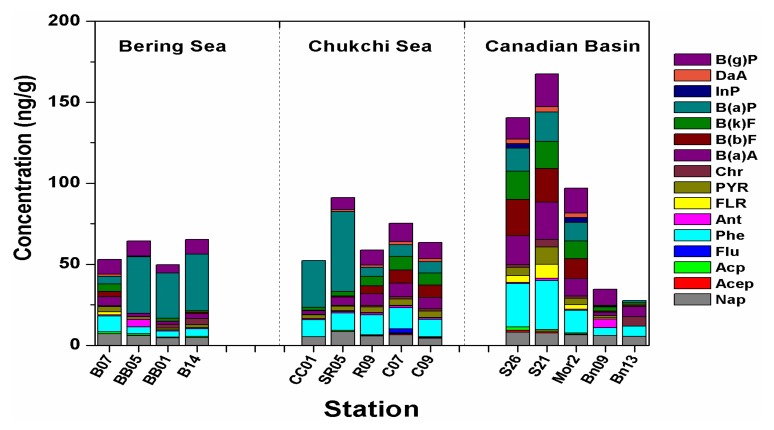
Concentrations (ng/g) of 16 PAHs compounds in sediments of Bering Sea, Chukchi Sea, and Canadian Basin.

Concentrations of the 16 US EPA prior PAHs in surface sediment samples in this study were presented in Figure 2 and Table S4. The concentrations of ∑PAHs ranged from 27.66 ng/g to 167.48 ng/g d.w. and the mean ∑PAHs was 77.27 ng/g. The levels of individual PAHs were in the range of undetectable to 49.34 ng/g. On average, B(a)P, Phe, and B(g)P were the most dominated compounds in this study. However, PAH compositions varied greatly with stations. For example, B(a)P were the highest component in shallow sites from the continental shelf of Bering and Chukchi Seas (station B14, BB01, BB05, CC01, and SR05). B(a)P is of great concern due to its high potentially carcinogenic toxicities. The reason why the level of B(a)P increased sharply in those five sites still needs further investigation since this area supports large bodies of commercial fish production. Phe was almost dominated in the other stations, with higher proportions of B(a)A, B(b)F, B(k)F, and B(a)P in the continental shelf breaks of the Chukchi Sea, where the highest ∑PAHs were located. These are dominated compounds in coal combustion emission [49]. It has been pointed out that the majority of the high molecular weight PAHs on the Beaufort Sea Shelf have a principal source in the Mackenzie River [50], which may also partly explain the highest value of PAHs in this region. There were also many individual PAHs undetectable in some stations. For example, Flu was only detected in station C07 with the level of 2.67 ng/g, while InP were merely detected in station S26 and Mor2 with the concentrations of 2.83 and 2.96. The different PAH patterns in the study area may be induced by a combination of PAH sources, geographic position, sediment properties (total organic carbon and granulometry), water depth, and sedimentation rates.

**Figure 3:**
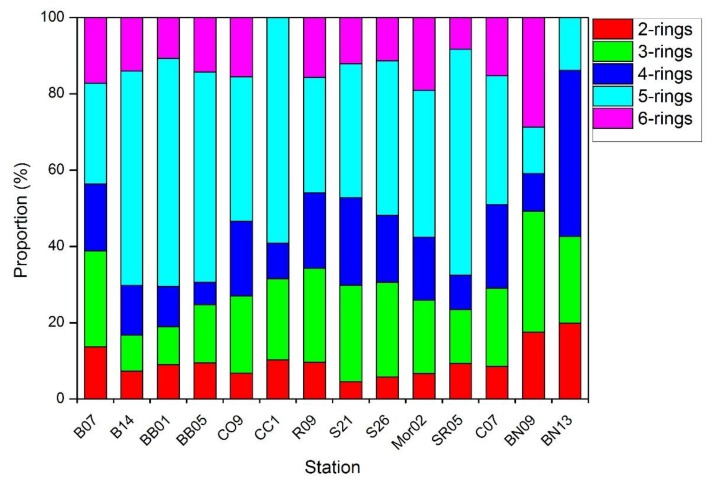
Contribution (%) of two-, three-, four-, five- and six-ring PAHs to total sum at studied sites.

Figure 3 illuminated the proportions of different ring PAHs at the study area. The results reflected that three-ring (4.94 ng/g to 42.24 ng/g) and five-ring (4.2 ng/g to 56.77 ng/g) PAHs were the most abundant compounds in all the stations. It should be noted that the proportion of five- and six-ring components were obviously lower in the Pacific and Canadian Basin (B07, Bn09, and Bn13), which may be due to the fact that PAHs with high log K_OW_ tended to absorbed strongly with particles and deposited nearby from their sources. This hindered their potential to conduct long-range transport to the remote oceans.

#### 3.2.2. Comparison with Other Places in Arctic Ocean

The investigations of PAHs in Arctic areas were scarce, because they are far away from human beings, and the harsh Arctic climate and environment create difficulties for sampling. As concern for environment has increased, the studies focused on Arctic areas have also increased gradually. However, previous studies mainly focused on coastal sites in Canada, Russia, and Norway which have relatively higher contamination levels. In order to evaluate the quality of sediment, the PAHs levels were compared with other remote areas (Table 1). However, our results are much lower than the data reported in other Arctic Ocean sediments like Beaufort Sea [51,52], Chukchi Sea [53], Chukchi shelf [54], Northern Ireland lake [55], Cork Harbor [11], two harbor in Norway [56], and the Ardal Fjords [57]. On the one hand, some of the high level regions were either closed to the continent or contributed by point sources. On the other hand, the difference may also be due to some of these studies taking alkyl-PAHs into consideration when calculating ∑PAHs, and the dominance of alkyl-PAHs has been reported in sediments from the Chukchi shelves [54]. In general, the PAHs levels in the study were comparative or slightly lower than those in Ob (24–115 ng/g), Yenisei (40–131 ng/g), Kara Sea (16–94 ng/g), White Sea (27–95 ng/g), and higher than those in Ny-Alesund (27–34 ng/g) [44,58,59] and the northern South China Sea (11.3–95.5 ng/g) [49] (Table 1). With the comparison of other regions around the world (Table 1), the concentration of sediment PAHs according to this study were at a relatively lower concentration range (<200 ng/g) which indicated that these areas were relatively non-polluted.

### 3.3. PAHs Spatial Distribution

The study area was divided into three regions: Bering Sea, Chukchi Sea, and Canadian Basin. The investigation (Figure 2) showed that the concentrations of ∑PAHs had a trend which decreased in the order: southern Canadian Basin > Chukchi Sea > Bering Sea, whereas the northern part of the Canadian Basin (stations BN09 and BN13) contained the lowest concentrations of PAHs. Pollutants are more difficult to get to the deep ocean in the higher Arctic compared with the continental shelf. In addition, the deposition rates vary in different areas of the sea, and surface sediment samples collected at a depth of 2 cm represent different ages. Therefore, the top 2 cm of surficial sediments collected in this study represent an accumulation period of 25 years in the Bering Strait and Chukchi shelf regions, 50 years in the Bering Sea, but a much longer period (>1000 year) in the Canada Basin and the central Arctic Ocean [13]. As such, in the deeper ocean sediments, a grab sample of 2 cm in depth will effectively ‘dilute’ the POPs associated with the upper sediment.

However, the extremely high levels of ∑PAHs were discovered at station S21 (167.48 ng/g) and S26 (140.4 ng/g), which were located in the edge of the continental shelf of the Chukchi Sea and dominated by Phe and B(b)F. In general, the reason why the contamination levels of the Canadian Basin margin region were the highest during this research may be attributed to an input of Canada point sources, atmospheric sedimentation, river runoff etc. For example, high-value stations (ΣPAHs > 100 ng/g) were distributed through Barrow Point and the Prudhoe Bay offshore area, close to the mouth of the Colleyville Rivers and Mackenzie River (account for 330 km^−3^ a^−1^ river outflow in the Arctic) [64]. Moreover, the clockwise flow of the Beaufort Gyre is conducive to the transportation of river-borne materials westward from the Beaufort Sea and along the Northwind Ridge to the northern Chukchi Sea [65]. Furthermore, the Siberian Coastal Water, which also brings pollutants from Yukon River, flow into the Eastern Chukchi Sea and finally turn into the Canadian Basin through the Chukchi Sea shelf, which also contributed to the accumulation of coastal pollutants in this area.

### 3.4. Potential Source of PAHs

Source apportionment analysis is becoming a routine in investigating the occurrences and accumulations of PAHs in natural environments. There are many methods that can be used to indicate the sources of PAHs, such as isomer ratios method, principle component analysis, chemical mass balance method, and stable carbon isotope method [66,67]. Relatively, isomer ratios were the most frequently used method to infer the sources of PAHs. They determine the possible PAH source by comparing the ratios of selected PAH compounds to the known ratios presented in specific sources. In this investigation, the isomer ratios of PAHs components, Phe/Ant, BaA/(BaA+Chy), and LMW/HMW were chosen to act as chemical tracers to examine possible sources of PAHs in this study. Based on the isomer parameters, the fuel oil/combustion transition point for Phenanthrene/Anthracene is 10, and LMW/HMW > 1 implies petroleum source and LMW/HMW < 1 indicates combustion origin. While BaA/(BaA+Chy) < 0.2 indicates a contribution of petroleum, BaA/(BaA+Chy) > 0.35 indicates the combustion contribution, and if it is between 0.2 and 0.35, it suggests petroleum combustion.

The three isomer ratios were displayed in Table S5. In the Bering Sea, BaA/(BaA+Chy) and LMW/HMW ratios were in the range of 0.46–0.91 and 0.20–0.63 while Phe/Ant ranged from 0.93–11.89, indicating combustion inputs combined with possible weaker petroleum. For the Chukchi Sea, ratios of Phenanthrene/Anthracene ranged from 9.27 to 13.11 indicating that PAHs were mainly originated from petroleum source. The ratios of LMW/HMW and BaA/(BaA+Chy) ranged from 0.31 to 0.52 and 0.84 to 0.95 respectively, indicating that PAHs were mainly derived from combustion source. For the Canadian Basin, Phenanthrene/Anthracene, BaA/(BaA+Chy) and LMW/HMW ratios varied from 11.49 to 34.70, 0.51 to 0.83, and 0.35 to 0.44. In all study stations, the BaA/(BaA+Chy) ratios were larger than 0.35, which indicated a contribution of combustion sources of PAHs. Analogously, as shown in Figure 3—6 ring PAHs were more abundant than 2–3 ring PAHs also figure out the combustion sources. Complementary, except station B14, BB01, BB05, R09, BN09, and BN13, the Phe/Ant > 10 in the rest stations implied an input of combustion contribution.

**Figure 4:**
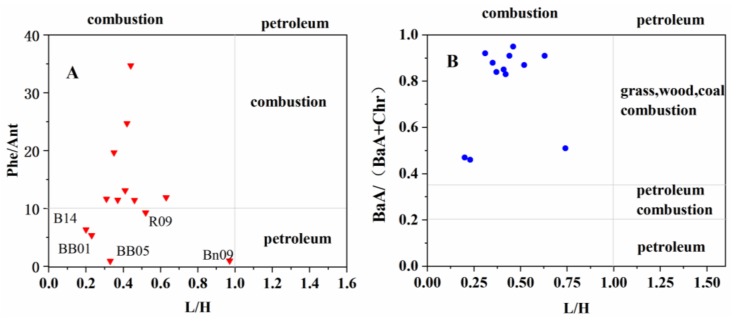
The use three PAH isomer pair ratios to identify the possible sources of PAHs in surface sediments of Bering Sea, Chukchi Sea, and Canadian Basin areas (red inverted triangles represent the value of Phe/Ant and L/H, while blue circles represent the ratios of BaA/(BaA+Chr) and L/H.

On the whole, the major source of the study areas was combustion inputs combined with possible weaker petroleum contribution (Figure 4). However, due to the lack of alkylated PAHs, it was hard to well assess the influence from petrogenic input, since the parent PAHs are largely derived from combustion processes. The conclusion for the source of PAHs in the Arctic is literature specific. The results here were similar with the research which pointed out that one of the important sources of PAHs depositions was coal combustion in east Canada [61]. Ding et al. (2007) [1] also proposed that coal combustion contributed largely to the PAHs over arctic atmosphere. Natural sources, such as summertime forest fires in subarctic parts of Alaska, Canada, Russia, and Siberia can result in the episodic input of PAHs and other pollutants to the Arctic atmosphere. It is reported that a series of wildfires broke out in Russia starting in late July 2010 [68]. Thus, these regional combustion sources may have affected PAH in Arctic Ocean sediment.

It should be noted that microbial degredation and photodegradation have effects on molecular ratios even though they were regularly used for identifying possible sources [69]. Furthermore, LMW PAHs can be biodegraded more quickly than HMW PAHs [70]; Compared with Chy, the half-life of BaA was shorter when exposed to atmosphere and sunlight [71]. PAHs may undergo photo-degradation reaction with OH radicals during atmospheric LRT. Therefore, the diagnostic ratios would have a deviation because of direct or indirect photolysis and should be used with caution [7].

### 3.5. Ecological Risk and Health Risk Assessment

Ecological risk assessment has been used as a practical tool to evaluate PAHs risks to ecosystems and living organisms. The procedures used for deriving the sediment quality guidelines (SQGs) are approach specific. In order to assess the impact that the PAHs have on aquatic ecosystems, Long et al. (1995) [72] and Macdonald et al. (1996) [73] put forward four sets of sediment quality guidelines (SQGs) into use, which is an acknowledged effective way to assess the ecological risk of ocean sediments.

We compared PAH concentrations from the sediment in these three areas with effects range-low value (ERL, the lower 10th percentile value of the effects data) and effects range-median value (ERM, 50th percentile of the effects data) [72], as well as probable effects level (PEL) and threshold effects level (TEL) [73]. These values are listed in Table S6. The results of comparison showed that all PAH concentrations from these sediment samples were lower than SQGs, which means that PAHs may have a low potential ecological risk to the surrounding life in northern Bering Sea, Chukchi Sea, and Canadian Basin.

To further assess whether the levels in the Arctic will pose harmful effect to the heath of organism and humans, health risk assessment is also necessary. In order to estimate an overall and individual carcinogenicity of PAHs, the total B(a)P toxic equivalent quotient (TEQcarc) for carcinogenic compounds was calculated to assess the health risk with the following equation

TEQ_PAH_ = ∑TEQ_i_ = ∑(C_i_ × TEF_i_) (1)

The toxic equivalency factors (TEFs) were used to quantify the carcinogenicity of other PAHs relative to B(a)P. The calculated TEF is 0.001 for NaP, Acep, Acp, Flu, Phe, FLR, and PYR; 0.01 for Ant, Chr, and B(g)P; 0.1 for BaA, BbF, BkF, and IP; 1 for BaP and DahA. The calculated TEQ ranged from 1.6–51.34 ng TEQ g^−1^ (Table S7). Specifically, the values of TEQ_PAH_ range from 7.59 ng TEQ g^−1^ to 35.72 ng TEQ g^−1^, 9.12 ng TEQ g^−1^ to 51.34 ng TEQ g^−1^ and 1.60 ng TEQ g^−1^ to 27.92 ng TEQ g^−1^ from Bering Sea, Chukchi Sea, and Canadian Basin. In these areas, Bering Sea has the highest TEQ_PAH_ mean value (26.79 ng TEQ g^−1^), although the highest concentrations actually occurred in the Canadian Basin. Canadian Council of Ministers of the Environment (CCME) has applied risk-based criterion for protection of environmental and human health (CCME 2010), indicating a safe level of 600 ng TEQ g^−1^ [54]. All the values of sediment samples are below this guideline.

## 4. Conclusions

The record of human impact on Bering Sea, Chukchi Sea, and Canadian Basin was assessed by the indicators polycyclic aromatic hydrocarbons (PAHs) in sediments, providing available information on their levels, distributions, potential sources, and risk assessment in the Western Arctic and subarctic Ocean. The concentrations of PAHs were relatively low, which might be due to the remote locations. Additionally, the concentrations of ∑PAH were highest in the margin edges of the Canadian Basin, which may result from an accumulation of Canada point sources and river runoff while. Three isomer ratios of PAHs (Phenanthrene/Anthracene, BaA/(BaA+Chy), and LMW/HMW) showed the main source of combustion combined with weaker petroleum combustion contribution. Compared with four sediment quality guidelines, the concentrations of PAH are much lower, indicating a low potential ecological risk. All TEQ_PAH_ also shows a low risk to human health and they were highest in Bering Sea, which indicated a necessity for health risk assessment for PAHs in sediment, as high levels did not reveal high TEQ_PAH_. Further studies should be carried out taking consideration of other factors, such as atmospheric effects, terrestrial input, deposition and mixing activity, as well as water/sediment interface flux, aiming at a better understanding of the controlling factors involved.

## Figures and Tables

**Table 1:** Comparison of PAHs levels in surface sediments from remote regions.

Region	Number	∑PAHs (ng/g d.w.)	Reference
Bering Sea	16	49.84–65.38	This Study
Chukchi Sea	16	52.40–91.25	
Canadian Basin	16	27.66–167.48	
Western Beaufort Sea (Alaska)	6	159–1092	[51]
NS&T Alaska	6	2.17–733	
Barent Sea	-	1500	[41]
Cork Harbour in south east in Ireland	21	924–2877	[11]
Northern Ireland lake	-	83~2300	[55]
Harbor (Oslo and Drammen), Norway	16	3000–4800	[56]
Ardal Fjords, Norway	13	45,565–784,296	[57]
Ny-Ålesund, Svalbard, Norway S1	15	34	[44]
Ny-Ålesund, Svalbard, Norway S2	15	27	
White Sea, Russia	27	27–95	[58]
White Sea, Russia	27	13–208	[58]
Ob	12	24–115	[59]
Yenisei	12	40–131	
Kara Sea	12	16–94	
Northern South China Sea	15	11.3–95.5	[49]
Fjord areas, Barents	28	209–326	[60]
Tromsoflaket, Barents	28	58.8–224	
Ingoydjupet, Barents	28	157–217	
Eastern Canada	13	48–2790	[61]
Western areas in Ireland	15	100–1422	[62]
Northeastern Alberta, Canada	35	ND-3900	[63]

## Supplementary Materials

The following are available online at http://www.mdpi.com/1660-4601/15/4/734/s1, Table S1: Information about the sampling stations, Table S2: The grain size of surface sediments from study areas, Table S3: The Pearson correlation of grain size of surface sediments and PAHs from study areas, Table S4: Summary of PAHs concentrations (ng/g d.w.) in the different study stations, Table S5: Ratios of LMW/HMW, Phe/Ant, and BaA/(BaA+Chy) in study areas; Table S6: Standard pollution criteria of PAHs for sediment matrixes, Table S7: Estimated human health risk caused by PAH contaminated sediment.
