# Supplementary material for: Occurrence and Risk Assessment of PAHs in Surface Sediments from Western Arctic and Subarctic Oceans
Source: Int J Environ Res Public Health. 2018 Apr 12;15(4):734. doi: 10.3390/ijerph15040734 (PMC5923776; doi:10.3390/ijerph15040734)
Supplement: Supplementary file 1 [file ijerph-15-00734-s001.pdf]

Supporting Information

MANUSCRIPT TITLE: Occurrence and risk assessment of PAHs in surface sediments from western Arctic and Subarctic Oceans

List of Contents

Table S1. information about the sampling stations..2
Table S2. The grain size of surface sediments from study areas. 3
Table S3. The Pearson correlation of grain size of surface sediments and PAHs from study areas...4
Table S4 Summary of PAHs concentrations (ng/g d.w.) in the different study stations. 5
Table S5. Ratios of LMW/HMW, Phe/Ant and BaA/(BaA+Chy) in study areas. 6
Table S6. Standard pollution criteria of PAHs for sediment matrixes. 7

Table S1. information about the sampling stations.

Areas	Stations	Longitude	Latitude	Depth (m)
Bering Sea	B07	176.20	58	3743
	B14	-177.69	60.92	130
	BB01	-177.48	61.29	130
	BB05	-175.33	62.54	79
Chukchi Sea	CC01	-168.96	67.67	50
	SR05	-169.00	69.00	54
	R09	-168.94	71.96	51
	C09	-159.71	71.81	50
	C07	-165.33	72.54	51
	S21	-154.72	71.62	46
Canadian Basin	S26	-153.55	72.70	3521
	Mor2	-158.99	74.55	1224
	BN09	-167.13	84.19	2500
	BN13	-176.63	88.39	3995

Table S2. The grain size of surface sediments from study areas.

		Clay （%）	Silt （%）	Gravel（%）	Sand （%）
B14	Bering Sea	8.28	73.83	16.80	1.09
BB01		7.91	69.81	21.24	0.99
BB05		7.48	71.36	20.05	1.11
B07		12.59	69.59	15.08	2.71
CC01	Chukchi Sea	4.80	62.32	29.97	2.93
Mor02		26.27	62.10	11.28	0.37
C07		12.61	75.40	11.11	0.86
R09		9.65	77.14	11.97	1.24
C09	Canadian Basin	12.13	71.31	14.33	2.23
S21		17.36	68.29	13.49	0.87
S26		22.94	72.38	4.69	0.00
SR05		6.06	76.13	16.07	1.75
BN09		38.33	50.93	10.34	0.42
BN13		37.05	56.06	6.81	0.10
Average		15.96	68.33	14.52	1.19

Table S3. The Pearson correlation of grain size of surface sediments and PAHs from study areas.

Pearson correlation					
	clay	silt	gravel	sand	PAHs
clay	1	-	-	-	-
silt	-0.776**	1	-	-	-
gravel	-0.698**	0.093	1	-	-
sand	-0.665**	0.249	0.710**	1	-
PAHs	-0.048	0.229	-0.166	-0.208	1

*Significant at $p<0.05$, **significant at $p<0.01$

Table S4 Summary of PAHs concentrations (ng/g d.w.) in the different study stations.

Areas	Stations	Nap	Acep	Acp	Flu	Phe	Ant	FLR	PYR	Chr	B(a)A	B(b)F	B(k)F	B(a)P	InP	DaA	B(g)P	∑PAHs
Bering Sea	B07	7.25	ND	1.12	ND	9.63	0.81	1.76	3.37	0.56	5.37	3.44	4.46	4.83	ND	1.30	9.14	53.03
	B14	4.81	ND	0.76	ND	4.63	0.73	ND	1.84	3.57	3.11	0.85	1.00	34.86	ND	ND	9.21	65.38
	BB01	4.51	ND	0.50	ND	3.75	0.70	ND	1.29	2.12	1.83	0.04	1.84	27.91	ND	ND	5.35	49.84
	BB05	6.09	0.20	0.83	ND	4.24	4.57	ND	1.60	ND	2.13	ND	0.15	34.86	ND	0.48	9.18	64.33
Chukchi Sea	CC01	5.36	ND	ND	ND	10.29	0.90	ND	2.25	0.13	2.50	0.19	1.90	28.88	ND	ND	ND	52.40
	SR05	8.51	ND	0.77	ND	10.71	0.92	0.46	2.81	0.46	4.99	0.84	2.77	49.34	ND	1.03	7.61	91.25
	R09	5.67	0.49	0.37	ND	12.15	1.31	0.17	3.50	1.07	7.09	4.99	5.58	5.70	ND	1.52	9.25	58.86
	C09	4.29	0.51	0.49	ND	10.69	0.93	0.21	3.98	1.34	7.08	7.65	7.42	7.11	ND	1.80	9.87	63.37
	C07	6.44	0.61	0.50	2.67	13.11	1.00	0.21	3.98	1.50	8.41	8.14	8.24	7.25	ND	1.76	11.53	75.34
Canadian Basin	S21	7.66	1.19	0.79	ND	30.39	1.23	8.64	10.71	4.62	23.12	20.68	16.68	18.30	ND	3.25	20.21	167.48
	S26	8.15	1.20	1.98	ND	26.72	0.77	4.11	5.09	1.81	17.86	22.36	17.51	13.99	2.83	2.91	13.12	140.40
	Mor2	6.48	0.49	0.54	ND	13.97	0.71	2.92	3.91	1.41	10.74	12.35	10.78	11.56	2.96	2.64	15.53	96.99
	BN09	6.04	ND	ND	ND	4.93	5.25	0.78	1.25	ND	2.14	0.76	2.55	0.42	ND	0.47	9.92	34.51
	BN13	5.49	ND	ND	ND	6.31	ND	ND	ND	5.85	6.18	0.99	1.28	1.57	ND	ND	ND	27.66

"ND": not detected.

Table S5. Ratios of LMW/HMW, Phe/Ant and BaA/(BaA+Chy) in study areas.

areas	stations	L/H	Phe/Ant	BaA/(BaA+Chy)
Bering Sea	B07	0.63	11.89	0.91
	B14	0.2	6.34	0.47
	BB01	0.23	5.36	0.46
	BB05	0.33	0.93	-
Chukchi Sea	CC01	0.46	11.43	0.95
	SR05	0.31	11.64	0.92
	R09	0.52	9.27	0.87
	C09	0.37	11.49	0.84
	C07	0.41	13.11	0.85
Canadian Basin	S21	0.42	24.71	0.83
	S26	0.44	34.7	0.91
	Mor2	0.35	19.68	0.88
	BN09	0.97	0.94	-
	BN13	0.74	-	0.51

Table S6. Standard pollution criteria of PAHs for sediment matrixes.

PAHs	Range (ng/g)	ERL	ERM	TEL	PEL	TEF
Nap	4.29-8.51	160	2100	34.6	391	0.001
Acep	nd-1.2	16	500	6	88.9	0.001
Acp	nd-1.98	44	640	-	130	-
Flu	nd-2.67	19	540	21.2	144	0.001
Phe	3.75-30.39	240	1500	86.7	544	0.001
Ant	nd-5.25	85	1100	46.9	245	0.001
FLR	nd-8.64	600	5100	113	1494	0.01
PYR	nd-10.71	665	2600	153	1398	0.001
Chr	nd-5.85	384	2800	108	846	0.01
B(a)A	1.83-23.12	261	1600	74.8	693	0.1
B(b)F	nd-22.36	-	-	-	-	0.1
B(k)F	0.15-17.51	-	-	-	-	0.1
B(a)P	0.42-49.34	430	1600	88.8	763	1
InP	nd-2.96	-	-	-	-	0.1
DaA	nd-3.25	63	260	6.2	135	1
B(g)P	nd-20.21	-	-	-	-	0.01

ERL effects range-low value, ERM effects range-median value, TEL threshold effect levels, PEL probable effect levels, TEF toxic equivalency factor.

Table S7 Estimated human health risk caused by PAH contaminated sediment.

		TEQi																TEQPAH
		Nap	Acep	Acp	Flu	Phe	Ant	FLR	PYR	Chr	B(a)A	B(b)F	B(k)F	B(a)P	InP	DaA	B(g)P	∑(g)P
		0.001	0.001		0.001	0.001	0.001	0.01	0.001	0.01	0.1	0.1	0.1	1	0.1	1	0.01	
Bering Sea	B07	0.00725	nd	0.00112	nd	0.00963	0.0081	0.00176	0.00337	0.0056	0.537	0.344	0.446	4.83	nd	1.3	0.0914	7.59
	B14	0.00481	nd	0.00076	nd	0.00463	0.0073	nd	0.00184	0.0357	0.311	0.085	0.1	34.86	nd	nd	0.0921	35.50
	BB01	0.00451	nd	0.0005	nd	0.00375	0.007	nd	0.00129	0.0212	0.183	0.004	0.184	27.91	nd	nd	0.0535	28.37
	BB05	0.00609	0.0002	0.00083	nd	0.00424	0.0457	nd	0.0016	nd	0.213	nd	0.015	34.86	nd	0.48	0.0918	35.72
Chukchi Sea	CC01	0.00536	nd	#VALUE!	nd	0.01029	0.009	nd	0.00225	0.0013	0.25	0.019	0.19	28.88	nd	nd	nd	29.37
	SR05	0.00851	nd	0.00077	nd	0.01071	0.0092	0.00046	0.00281	0.0046	0.499	0.084	0.277	49.34	nd	1.03	0.0761	51.34
	R09	0.00567	0.00049	0.00037	nd	0.01215	0.0131	0.00017	0.0035	0.0107	0.709	0.499	0.558	5.7	nd	1.52	0.0925	9.12
	C09	0.00429	0.00051	0.00049	nd	0.01069	0.0093	0.00021	0.00398	0.0134	0.708	0.765	0.742	7.11	nd	1.8	0.0987	11.27
	C07	0.00644	0.00061	0.0005	0.00267	0.01311	0.01	0.00021	0.00398	0.015	0.841	0.814	0.824	7.25	nd	1.76	0.1153	11.66
Canadian Basin	S21	0.00766	0.00119	0.00079	nd	0.03039	0.0123	0.00864	0.01071	0.0462	2.312	2.068	1.668	18.3	nd	3.25	0.2021	27.92
	S26	0.00815	0.0012	0.00198	nd	0.02672	0.0077	0.00411	0.00509	0.0181	1.786	2.236	1.751	13.99	0.283	2.91	0.1312	23.16
	Mor2	0.00648	0.00049	0.00054	nd	0.01397	0.0071	0.00292	0.00391	0.0141	1.074	1.235	1.078	11.56	0.296	2.64	0.1553	18.09
	BN09	0.00604	nd	nd	nd	0.00493	0.0525	0.00078	0.00125	nd	0.214	0.076	0.255	0.42	nd	0.47	0.0992	1.60
	BN13	0.00549	nd	nd	nd	0.00631	nd	nd	nd	0.0585	0.618	0.099	0.128	1.57	nd	nd	nd	2.49
